# Applicability of non-invasively collected matrices for human biomonitoring

**DOI:** 10.1186/1476-069X-8-8

**Published:** 2009-03-09

**Authors:** Roel Smolders, Karl-Werner Schramm, Marc Nickmilder, Greet Schoeters

**Affiliations:** 1 VITO, Environmental Toxicology, Boeretang 200, 2400 Mol, Belgium; 2 Helmholtz Zentrum München, German Research Center for Environmental Health, Ingolstaedter Landstr. 1, 85764 Neuherberg, Germany; 3 Université Catholique de Louvain (UCL), Unité de Toxicology Industrielle et de Medicine du Travail, Clos Chapelle-aux-Champs 30–54, 1200 Brussels, Belgium

## Abstract

With its inclusion under Action 3 in the Environment and Health Action Plan 2004–2010 of the European Commission, human biomonitoring is currently receiving an increasing amount of attention from the scientific community as a tool to better quantify human exposure to, and health effects of, environmental stressors. Despite the policy support, however, there are still several issues that restrict the routine application of human biomonitoring data in environmental health impact assessment. One of the main issues is the obvious need to routinely collect human samples for large-scale surveys. Particularly the collection of invasive samples from susceptible populations may suffer from ethical and practical limitations. Children, pregnant women, elderly, or chronically-ill people are among those that would benefit the most from non-invasive, repeated or routine sampling. Therefore, the use of non-invasively collected matrices for human biomonitoring should be promoted as an ethically appropriate, cost-efficient and toxicologically relevant alternative for many biomarkers that are currently determined in invasively collected matrices. This review illustrates that several non-invasively collected matrices are widely used that can be an valuable addition to, or alternative for, invasively collected matrices such as peripheral blood sampling. Moreover, a well-informed choice of matrix can provide an added value for human biomonitoring, as different non-invasively collected matrices can offer opportunities to study additional aspects of exposure to and effects from environmental contaminants, such as repeated sampling, historical overview of exposure, mother-child transfer of substances, or monitoring of substances with short biological half-lives.

## Background

The recent adoption of human biomonitoring (HBM) as Action 3 in the Environment and Health Action Plan 2004–2010 of the European Commission [1,2] has motivated the implementation and application of HBM in European environment and health research.

One of the most important issues hampering the routine application of HBM on a large scale is the obvious need to collect human samples, often invasively. Blood has the undeniable advantage that it is in contact with all tissues and in equilibrium with organs and tissues. Therefore, it has been used extensively for various research and survey goals. However, blood sampling is an invasive procedure and suffers from ethical and practical constraints, particularly for small children or other susceptible populations [3,4]. The European Commission's SCALE Initiative (Science, Children, Awareness, EU Legislation and continuous Evaluation) has specifically identified children as a main target population for environment and health policies, so including this subpopulation in any HBM project is a priority [5]. Likewise, sampling non-invasively collected matrices is preferable in particularly vulnerable groups, such as pregnant women, elderly, or chronically-ill people.

Additionally, repeated or even routine biomonitoring may be desirable for the efficiency evaluation of risk management options and efficacy of environment and health policies. For short-lived chemicals such as volatile organic compounds or agricultural pesticides, average exposures may not reflect peak exposures arising through infrequent exposure episodes. Repeated sampling of high-exposure subjects provides more insight into the true nature of these episodes and of their toxicological consequences [6,7].

Finally, because non-invasively collected matrices need less specialized personnel for sampling, costs associated with large sampling designs may be significantly reduced [8,9].

**Figure 1:**
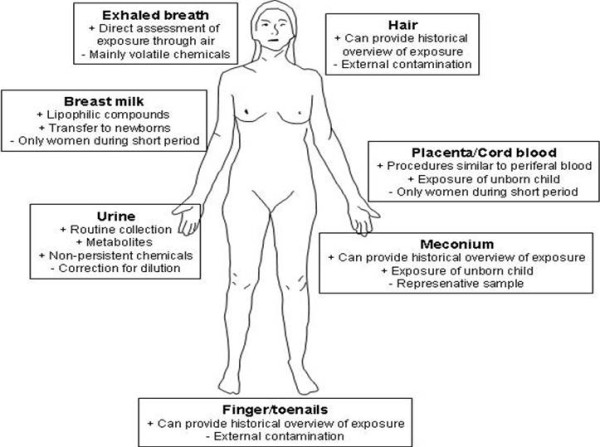
**Some properties of different non-invasively collected matrices for routine human biomonitoring application**.

Because of these advantages, there is a strong case for non-invasively collected matrices for human biomonitoring as an ethically appropriate, cost-efficient and toxicologically relevant alternative for many of the biomarkers currently determined in invasive matrices. Moreover, a well-informed choice of matrix can provide an added value for HBM, as different non-invasively collected matrices offer opportunities to study additional aspects of exposure to and effects from environmental contaminants, such as short- and long-term toxicokinetics, changing exposure over time, or monitoring of volatile chemicals or substances with short half-lives (Figure 1) [4].

### An overview of non-invasively collected matrices

It has been shown extensively that even the unborn child may already prenatally come in contact with hazardous substances [4,10]. While the placenta is the obvious choice to study this exposure pathway, it can also act as a barrier for toxicants and thereby to some extend reduce exposure of the foetus [11,12]. Using placenta and/or cord blood as a matrix for biomonitoring offers the advantage that at the same time, the exposure history of both the mother and the early exposure of the newborn infant is studied. A downside of using the placenta as a biomonitoring matrix, is that it may be difficult to collect representative samples. It is a complex mixture of blood vessels, chorionic villi and membranes, and metals for example are not uniformly distributed [12,13]. Cord blood has the additional advantage that several well-defined and documented standard operation procedures (SOPs) for peripheral blood can be applied. However, the volume of cord blood samples is usually limited and collection suffers from the major disadvantage that it is not always practically feasible to collect repeated representative samples [14]. The main care during delivery goes to the well-being of mother and child, and collecting cord blood samples may not always be practically feasible.

Also after birth, newborns may take up chemicals through specific pathways that offer great potential as non-invasively collected matrices for biomonitoring. Human milk is considered one of the most acceptable matrices for monitoring persistent bio-accumulating toxicants (PBTs). In the lactation period, human milk is a major uptake route for environmental contaminants due to its fatty character, especially for primigravidae mothers [15,16]. Human milk is easy to collect, enriched in lipophylic compounds and represents the main exposure source for breast feeding infants [17]. One of the major disadvantage of using placenta, cord blood or human milk as a non-invasively collected matrix for biomonitoring is that only women and their children can be included in a biomonitoring study, and only at certain periods in their lives [3].

Urine is probably the most frequently used matrix in humans to quantify the degree of environmental or occupational exposure to pollutants, especially for substances with short biological half-lives [3,18]. The collection and analysis of urine samples carries no associated risk, and large volumes can at once be gathered per individual [19]. Spot collection of samples is most frequently used in biomonitoring programmes, especially for surveys where large numbers of samples need to be gathered. The major disadvantage of using spot samples is the variability in the volume and concentration of urine. Hence, spot urine samples are standardised based on two different methods:

• By expression per gram of creatinine [19,20]. The World Health Organisation (WHO) has developed guidelines which stipulate that samples with creatinine concentrations <30 or >300 mg/dL are regarded as either too diluted or too concentrated [21]. However, these guidelines have been questioned recently based on detailed assessment of the role of age, gender and ethnicity and may not be appropriate for pregnant women or children [18];

• Another commonly used method to standardise biomarker measurements in urine is to take account of the gravity or relative density of urine [19].

Probably the major advantage of urine as a non-invasively collected matrix is the ease with which repeated sampling can be performed without major ethical or practical limitations across all layers of the population, including babies, young children and other susceptible populations.

The advantage of repeated sampling is also valid when using hair as a non-invasively collected matrix. Hair has been successfully used to measure both internal and external exposure to a wide variety of organic and inorganic pollutants [22-24]. As hair grows about one centimetre per month, analysis of hair of different length may reflect cumulative exposure over several months. Taking advantage of this property of hair, differences in exposure over several months or even years can be followed. Potential constraints on the use of hair include the difficulty in differentiating between internal and external sources of contaminant and the widespread use of hair treatment products [25,26]. Also, repeated hair sampling is not always well-received for esthetical reasons, as this may impact hair style. Remarkably, also home-collection of urine samples may face some resistance, as some age groups are reluctant to walk around with their urine samples (E. den Hond, personal communication).

Using exhaled breath as a non-invasively collected matrix offers the potential to directly relate substances in ambient air to exhaled concentrations of biological and toxicological relevance. Measuring exposure and effect biomarkers in exhaled air may be highly suitable for the characterization of dose at the target organ level, in this case the lungs and respiratory system [27,28]. Because for most collection systems only tidal breathing is needed, the samples can be collected in a broad range of subjects. Samples can also be collected from very young children or individuals with airway diseases.

Also other matrices, such as meconium, finger/toenails or saliva offer great potential for routine application in human biomonitoring. Although their use and applicability is much less documented in literature than the ones previously discussed, some examples and potential future applications are given below.

Troublesome sample collection and level of invasiveness are commonly cited reasons why study participants do not want to provide biospecimens for research purposes [9]. Using non-invasively collected matrices, possibly gathered through home-collection, may dramatically improve participation rates of populations because less effort is involved for the participants [29,30] or because it lowers the guardians' objections for children's participation [31]. Improving the participation rates may have the added benefit that there is much less selection bias as a larger subsection of the general population will participate [32].

### Biomarkers of exposure

The application of different non-invasively collected matrices for the detection and quantification of environmental exposure to metals has abundantly been described in literature. Iyengar and Rapp illustrate how the placenta can be used to detect the presence of toxic trace elements such as arsenic, mercury or lead [13,33-35]. Others additionally provide data indicating that concentrations of trace elements can vary 5- to 10-fold due to specific exposure situations, such as living in the vicinity of coal and metal mining and smelting operations [36]. Also urine, finger/toenails, or human milk have repeatedly been used as suitable matrices of exposure for metals. [37-41]. Probably the best-known use of hair as a non-invasive matrix for metals is in the biomonitoring of organic and inorganic mercury, as hair is by far the best integrator of past exposure [42,43], although also other metals have repeatedly been monitored using hair [44-46].

Also for organic compounds, several non-invasively collected matrices have frequently been used to quantify chemicals that are lipophylic and resistant to metabolic degradation. Many persistent environmental pollutants, such as polychlorinated dibenzo-p-dioxins (PCDDs) and dibenzofurans (PCDFs) are widespread and can be found in placenta, cord blood, or human milk [47-53]. Meconium has been promoted as a very useful non-invasively collected matrix to describe exposure of the unborn child to pesticides or metals [54,55].

Urine has frequently been used as a matrix for a wide variety of both organic and inorganic compounds for occupational as well as environmental exposure [19,56,57]. As urine is a main excretory pathway, it is a preferred matrix to monitor for non-bioaccumulating and rapidly metabolised compounds. Interpretation of urinary biomarkers is sometimes complicated as they primarily focus on quantification of metabolites rather than the parent compounds. Some chemicals, like chlorpyrifos, are broken down in the body into a number of metabolites which can be detected in urine. As these same metabolites also occur as natural products of environmental degradation, it is not always possible to distinguish exposure to the parent compound from exposure to its environmental degradates [3,14].

The gaseous fraction of exhaled breath can be a good biomonitor for a wide variety of volatile substances [58,59]. The monitoring of metals in exhaled breath is less well documented, although Mutti et al [60] show that toxic metals and transition elements are detectable in exhaled breath condensate (EBC). This may have potential in the assessment of the target tissue dose for substances with potential pneumotoxic activity, such as Cd, Co or Ni [28,60]. A disadvantage of measuring biomarkers of exposure in the gaseous phase of exhaled air is that the residence time of substances are generally rather short, in the order of minutes or hours [58].

When using hair and finger/toenails to quantify exposure to environmental contaminants, caution is needed in the interpretation of exposure data. Many contaminants have been proven to reach hair and finger/toenails via two major routes: endogenous (xenobiotics reach the hair matrix through blood) and exogenous (atmospheric deposition) [3,23]. Hence it may be difficult to distinguish between contaminants taken up and those related to external contamination. Findings by Nakao et al [61] and Stupar et al [62] show that hair can in fact be used for the quantification of exogenous atmospheric exposure and in some cases even for the estimation of corresponding air concentrations. Mainly for substances where environmental exposure does not generally occur through air, the constraint of external contamination is limited. For methylmercury for example, which is generally taken up through food or dental amalgam, hair is by far the preferred matrix for biomonitoring and large-scale biomonitoring campaigns have clearly linked methylmercury with neurodevelopmental deficiencies [42]. While washing procedures have been shown to differentiate between endogenous and exogenous exposure, caution remains necessary in the interpretation of using hair as a non-invasively collected matrix for exposure [63].

Tables 1 and 2 provide an overview of typical concentrations of both metals and organic contaminants detected in different non-invasively collected matrices.

**Table 1:** An overview of typical concentrations of toxic trace metals reported in non-invasive human matrices (arithmetic mean (range^a^))

	As	Cd	Hg (total)	Pb
Urine (μg/l)	6.4 (ND-157) [37]	0.34 (ND-31.5) [37]	0.89 (ND – 34.8) [37]	1.3 (0.1–4.6)^b^ 0.8 (0.02–4.8)^c^ [38]
Urine (μg/g creatinine)	4.9 (ND-163) [37]	0.27 (ND-22.4) [37]	0.59 (ND-16.0) [37]	0.8^d ^(0.2–3.4)^b^ 0.5^d ^(0.1–4.6)^c^ [38]
Placenta (ng/g wet weight)	6 (3–12) [13]	4 (1–6) [13]	8 (2–13) [13]	34 (5–60) [13]
Cord blood (μg/l)	15.7 (2.9–74.6) [33]	0.02 (ND-0.08) [34]	10 (ND-75) [35]	11.2 (0.9 – 122) [34]
Exhaled breath (μg/l)	-	- (ND-1.70) [60]	-	- (ND-1.4) [60]
Breast milk (μg/l)	0.3 (0.1–0.8) [41]	0.1 (0.1–3.8) [41]	2.7 (0.64–257.1) [41]	5 (ND-41.1) [41]
Hair (μg/g)	0.65 (0.2–8.2) [44]	0.08 (ND-8.19) [45]	0.2 (0.04–1.73)^e^ [46]	2.26 (ND-583.5) [45]

^a ^Range may include high levels at which adverse effects are expected; ^b ^Data for children; ^c ^Data for adults; ^d ^Geometric mean; ^e ^Mean (P_10_-P_95_); ^f ^ND: below detection limit

**Table 2:** An overview of typical concentrations of toxic organic compounds reported in non-invasive human matrices (arithmetic mean (range^a^))

	Hexachlorobenzene (HCB) (ng/g lipid)	PCDD/F (in pg TEQ/g lipid)	Cotinine (in non-smokers)
Urine (μg/l)	PCP: 3.8 (0.6–18.0)^c^ PCBT: 8.8 (0.5–86.9)^c^ [57]	-	17.9 (ND-3400) [37]
Urine (μg/g creatinine)	-	-	17.1 (ND-5810) [37]
Placenta (ng/g wet weight)	7.7^b ^(2.2–26.5) [49]	31 (10–74) [47]	-
Cord blood (μg/l)	0.73 (0.14–9.82) [50]	14 (3.7–32) [47]	3.08 (ND-910) [51]
Exhaled breath (μg/l)	-	-	21 (ND-42)^e^ [59]
Breast milk (ng/g lipid)	12.4 (6.01–24.56) [49]	9.5 (2.7–51.5) [52]	0.2 (0.03–1.3) [53]
Hair (μg/g)	28 (20–32)^d^ [89]	- (0.25–230) [26]	0.29 (ND-11) [45]

^a ^Range may include high levels at which adverse effects are expected; ^b ^Arithmetic mean (P_10_-P_95_); ^c ^Metabolites of HCB, expressed in μg/24 h; ^d ^Median (P_25_-P_75_); ^e ^Cotinine in saliva; ^f ^ND: below detection limit

### Biomarkers of effect

Detecting the early hazardous effects of environmental contaminants has a high priority in the protection of neonates and newborn children [4]. In both placental tissue and cord blood, biomarkers for DNA damage have been measured using different methods, including ^32^P-postlabeling and enzyme-linked immunosorbent assay (ELISA) methods [64,65]. Although the estimated polycyclic aromatic hydrocarbon (PAH) dose to the foetus may be 10-fold lower than in the mothers because of the earlier mentioned barrier effect of the placenta, the PAH adduct levels in the newborns are similar or higher than those in their mothers [66]. This may imply that the foetus is far more susceptible to DNA damage than its mother [67]. Maervoet et al [68] outlined that, while there are still many gaps in the understanding of the relationship between environmental contaminants in cord blood and the functioning of the thyroid system, any interference may adversely affect neonatal neurodevelopment. The discovery that placental nucleic acids can be used as a marker for prenatal screening has recently opened the door for the application of rapidly developing technologies such as toxicogenomics and proteomics. This in the future may lead to the development of molecular markers for non-invasive prenatal gene expression profiling of the foetus using placenta and cord blood [69].

Also for other non-invasively collected matrices, the use of relatively new '-omics' technologies has opened the door for the detection of new biomarkers of effect. Recently, there has been increased research interest in the metabolic profiling (metabonomics or metabolomics) of mainly urine and exhaled breath samples. This technique encompasses the systematic profiling of metabolite concentrations and their systematic and temporal changes through effects from diet, lifestyle, environment and genetic [70,71]. Although metabonomics currently is not yet sufficiently developed for large-scale biomonitoring studies, it may be a promising tool for the rapid screening of metabolites [71,72].

Other non-invasively collected matrices already have a long-standing and well-documented history as a matrix in routine, often clinical, health assessment [73]. For example, α_1_- and β_2_-microglobulin excretion and retinol-binding proteins in urine have frequently been described as sensitive biomarkers of renal disfunctioning [74]. Additionally, urine has been used as a non-invasive matrix for the quantification of base DNA-adducts as biomarkers for carcinogenesis [75]. Urinary 8-hydroxy-deoxyguanosine (8-OHdG) has been used as a biomarker of the DNA repair response to oxidative stress and DNA-damaging compounds [14]. Exhaled breath has received much attention as a suitable clinical matrix for the early detection of pulmonary and respiratory diseases. It has extensively been demonstrated that fractional exhaled nitric oxide (FeNO) concentrations in exhaled air are higher in people suffering from various pulmonary diseases, including asthma and chronic obstructive pulmonary disease (COPD) [76,77]. Other biomarkers, such as exhaled breath temperature, pH of EBC, and the presence of cytokines (e.g. interleukins (IL) IL-4, IL-6, IL-8 and tumor-necrosis factor α (TNF-α)), 8-isoprostane or hydrogen peroxide may also be biomarkers of lung inflammation and oxidative stress [76,78]. Saliva as a non-invasively collected matrix has been used to quantify cholinesterase activity, a biomarker for potential neurotoxic effects [79,80]. Additionally, saliva specimens have been used in combination with the Ames test or a Chinese hamster V79 lung fibroblast cell line to investigate the genotoxic effects of smoking and alcohol consumption. exfoliated buccal cells have been used to monitor genetic damage in humans using the micronucleus test [81,82].

For other non-invasively collected matrices, such as hair, human milk, finger/toenails or meconium, we found no examples of biomarkers of effect.

### Correlations among (non-invasive collected) biomonitoring matrices

Many publications have measured and compared values for biomarkers of exposure for the same chemicals in different (non-invasively collected) matrices. Often, there are good correlations among invasively collected and non-invasively collected matrices, although several exceptions can be noticed [3].

In several cases, meconium has been shown to be the most sensitive matrix to analyse fetal exposure to environmental contaminants. For example in mercury or pesticides monitoring, meconium showed the highest exposure rates compared to cord blood and infant hair [54,83]. For phthalate metabolites, urinary concentrations have been found to be more informative than blood, serum or milk concentrations is the Swedish population. Urinary concentrations showed lower day-to-day variability and were detected at much higher concentrations than in other matrices [84]. For other contaminants, good correlations are often found between paired cord blood or placenta samples and other matrices such as maternal blood, human milk or amniotic fluid [85,86].

Urinary biomarkers generally reflect metabolites of compounds rather than the pure compound itself. This makes it more difficult to correlate urinary biomarkers with biomarkers of exposure in other matrices. Mainly for non-metabolised compounds, correlations between concentrations in urine and other matrices such as blood appear to be good [87]. Many authors also have addressed the correlation between contaminants in hair and other matrices [88,89]. For inorganic compounds, Mehra and Juneja [90] reported significant correlations between hair and fingernail concentrations of Cd and Pb, Stupar et al [62] found significant correlations between Pb in hair and blood, and Ng et al [91] reported significant correlations between hair mercury and mercury in blood, 24 h urine or cord blood samples following a meta-analysis of mercury biomonitoring studies.

Compared to invasively collected matrices such as peripheral blood, there are very little, if any, chemicals that cannot be determined with sufficient sensitivity in non-invasively collected matrices. Concentration of chemicals in non-invasively collected matrices generally correlates very well with other, often invasively collected, matrices such as peripheral blood.

### Validation status

Validation of biomarkers is a lengthy and difficult process, that requires the fulfilment of multiple criteria. In general, these criteria include understanding the biological and temporal relevance, pharmacokinetics, background variability, exposure-dose or dose-response relationship and identification of confounding factors [92].

Several urinary biomarkers have been fully validated for organic and inorganic substances, reference materials and internationally recognised standard methodologies are often available, and sampling procedures are well-documented. It is a widely-employed non-invasively collected matrix in many of the largest environmental studies such as the German Environmental Survey for children (GerES) [93], the American National Health and Nutrition Examination study (NHANES) [18], or the Flemish Human Biomonitoring programme [94]. Also human milk as a matrix has widespread use for biomarkers of exposure. It is easily collectable and fully validated for a wide variety of substances and reference materials. Internationally recognised standards for analysis are often available, and sampling procedures are well documented. The WHO coordinates a regular exposure study to using human milk to quantify the global presence and distribution of persistent organic pollutants. Within the context of this repeated human biomonitoring program, guidelines for developing a national protocol for sampling human milk have been developed [95,96]. Methods of collecting and sampling exhaled breath biomarkers include Tedlar bags, canisters, or even portable real-time detectors. These methods are generally well validated and documented, as are the measurement techniques [97]. Both the European Respiratory Society (ERS) and American Thoracic Society (ATS) have developed recommendations the collection and measurements in EBC and for measuring FeNO [76,77]. Because of the frequent application of meconium for illicit drug screening, structured screening protocols have also been developed for this non-invasively collected matrix [98,99].

A review by the Agency for Toxic Substances and Disease Registry highlighted some of the shortcomings of hair as a non-invasive biomonitoring matrix [25]. Although the review recognised that hair is a very useful matrix for identifying historical exposure to contaminants and may have predictive value towards health effects, a large amount of uncertainty remains regarding sampling procedures, quality assessment and control issues, and the lack of reference ranges and dose-response outcomes. Among others, hair colour and the use of hair treatment products may have a significant impact on the concentration and availability of chemicals [25,26]. Other matrices are often used in a research setting rather than a routine survey setting. While there may not be internationally accepted standard operation procedures available, confounding factors for several less frequently studied matrices are known. Slotnick et al [39] evaluated the effect of demographic characteristics and nutritional measures on the association between drinking water and toenail arsenic concentrations. Apart from toenail iron concentration, no demographic or nutritional parameters affected the biomarker response. In Kenya however, Were et al [100] reported that socio-economic background, health conditions, dietary habits and urban or rural living had a significant effect on lead, cadmium, zinc and iron concentrations in the fingernails of school children. For arsenic, Brima and co-workers illustrated that different ethnic groups showed significantly different concentrations of total arsenic in fingernails, suggestive of a different pattern of arsenic metabolism in different ethnic groups [101].

## Conclusion

For many years, blood has been seen as the ideal matrix for HBM as it is in contact with all tissues and in equilibrium with organs and tissues. However, blood sampling is an invasive procedure and suffers from ethical and practical constraints, particularly for small children or other susceptible populations. The use of non-invasively collected matrices can be a valuable alternative to, or addition for, invasive matrices for most contaminants discussed. Generally, there is good agreement between biomarkers measured in invasively and non-invasively collected matrices. However, if chosen carefully, non-invasively collected matrices can offer valuable added information on the evolution of exposure (meconium, hair, finger/toenail), the transfer of contaminants between mother and child (placenta, cord blood, breast milk) or the presence of volatile or rapidly metabolised substances (saliva, exhaled air, urine). By far the most advantageous property of non-invasively collected matrices is that repeated sampling, even in susceptible populations, poses a much lower stress on the participants and allows for a much wider inclusion of all layers of the population.

## Abbreviations

8-OHdG: 8-hydroxy-deoxyguanosine; ATS: American Thoracic Society; COPD: chronic obstructive pulmonary disease; EBC: exhaled breath condensate; ELISA: enzyme-linked immunosorbent assay; ERS: European Respiratory Society; FeNO: fractional exhaled nitric oxide; GerES: German Environmental Survey for Children; HBM: human biomonitoring; HCB: hexachlorocyclobenzene; IL: interleukine; INTARESE: Integrated Assessment of Health Risk of Environmental Stressors in Europe; ND: Below detection limit; NHANES: National Health and Nutrition Examination; PAH: polycyclic aromatic hydrocarbon; PBT: persistent bio-accumulating toxicants; PCBT: pentachlorobenzenethiol; PCP: pentachlorophenol; PCDD: polychlorinated dibenzodioxin; PCDF: polychlorinated dibenzofuran; SCALE: Science, Children, Awareness, EU Legislation and Continuous Evaluation; SOP: Standard operation procedure; TEQ: toxic equivalent; WHO: World Health Organisation.

## Competing interests

The authors declare that they have no competing interests.

## Authors' contributions

KWS was responsible for the section on hair and breast milk, MN provided information for the section on exhaled breath. RS and GS are responsible for the other chapters. All authors read and approved the final manuscript.
